# Supplementary material for: Ssu72 phosphatase is essential for thermogenic adaptation by regulating cytosolic translation
Source: Nat Commun. 2023 Feb 25;14:1097. doi: 10.1038/s41467-023-36836-y (PMC9968297; doi:10.1038/s41467-023-36836-y)

Supplementary Information

Ssu72 phosphatase is essential for thermogenic adaptation by regulating cytosolic translation

Eun-Ji Park[1,6], Hyun-Soo Kim[1,6,*], Do-Hyoung Lee[1], Su-Min Kim[1], Joon-Sup Yoon[1], Ji-Min Lee[2], Se Jin Im[3], Ho Lee[4], Min-Woo Lee[2,*], and Chang-Woo Lee[1,5,*]

Supplementary Figures 1-11

Supplementary Tables 1-2

Supplementary References

Uncropped gels and blots for Supplementary Figures

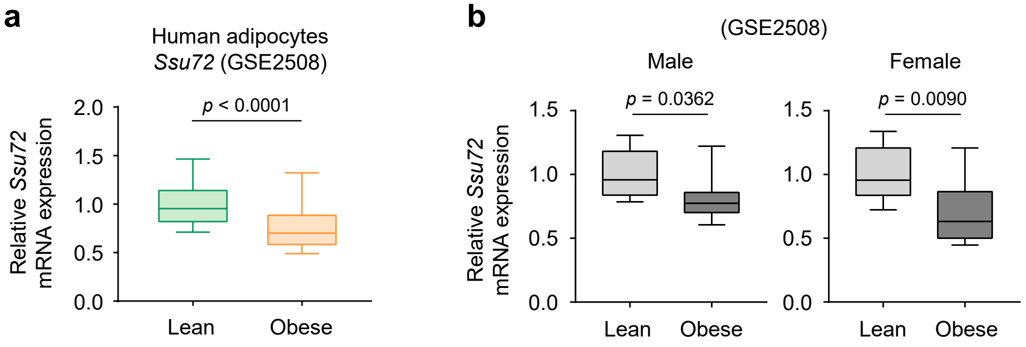

Supplementary Fig. 1: Clinical significance of Ssu72 expression in adipose tissue. a,b, Comparison of Ssu72 mRNA expression in adipocytes isolated from subcutaneous adipose tissue between non-diabetic lean ($n = 20$) and non-diabetic obese subjects ($n = 19$) (GEO accession number: GSE2508) (**a**), or comparison of *Ssu72* expression between lean and obese subjects in male and female groups, respectively (lean male, n = 10; obese male, n = 9; lean female, n = 10; obese female, n = 10) (**b**). Box-plots show respective medians (horizontal lines), 25th to 75th percentiles (boxes), and min-to-max ranges (whiskers). Data are presented as mean ± SEM. Statistical comparisons were made using two-tailed unpaired Student's *t*-test. Source data are provided as a Source Data file.

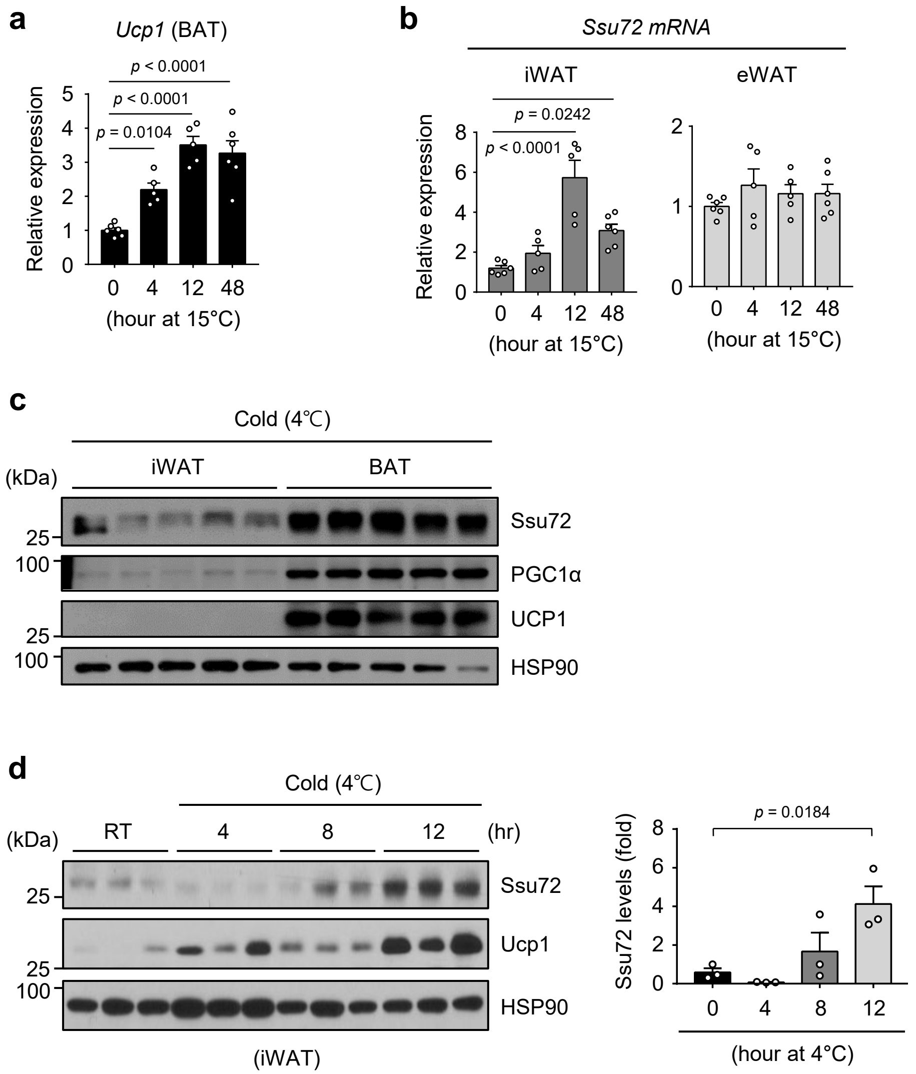

Supplementary Fig. 2: Ssu72 expression is induced by cold exposure in thermogenic adipose tissues. a,**b**, Relative mRNA expression levels of *Ucp1* in BAT (**a**) and *Ssu72* in iWAT and eWAT (**b**) of 7 to 8-week-old C57BL/6J male mice after mild cold exposure (15°C) (n = 5 mice per group). Data are presented as mean ± SEM. Statistical comparisons were made using one-way ANOVA. **c**, Western blots of lysates of iWAT and BAT from C57BL/6J male mice exposed to acute cold (4°C) for 4 hours (n = 5 mice per group). **d**, Protein expression levels of Ucp1 and Ssu72 in iWAT from C57BL/6J male mice housed at room temperature (RT, 23°C) and exposed to cold (4°C) for 4, 8, and 12 hours (n = 3 mice per group) (left), with quantification of Ssu72 protein levels normalized to HSP90 level using ImageJ (right). Data are presented as mean ± SEM. Statistical comparisons were made using one-way ANOVA. Source data are provided as a Source Data file.

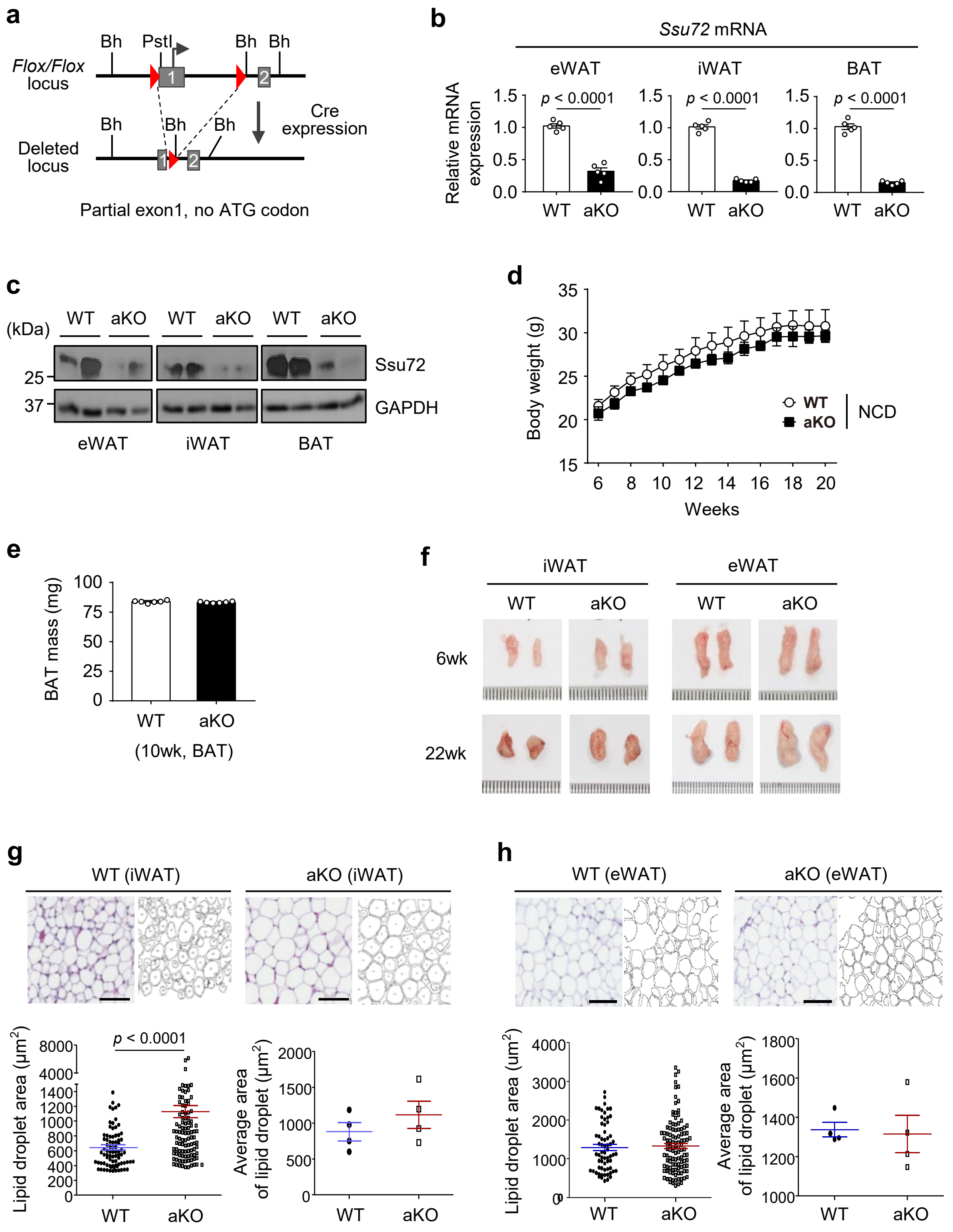
a
Flox/Flox locus
Bh
PstI
Bh
Bh
Cre expression
Deleted locus
Partial exon1, no ATG codon
b
Ssu72 mRNA
eWAT
iWAT
BAT
Relative mRNA expression
p < 0.0001
WT
aKO
c
(kDa)
WT
aKO
Ssu72
GAPDH
eWAT
iWAT
BAT
d
Body weight (g)
Weeks
WT
aKO
NCD
e
BAT mass (mg)
WT
aKO
(10wk, BAT)
f
iWAT
eWAT
WT
aKO
6wk
22wk
g
WT (iWAT)
aKO (iWAT)
Lipid droplet area (μm2)
p < 0.0001
Average area of lipid droplet (μm2)
WT
aKO
h
WT (eWAT)
aKO (eWAT)
Lipid droplet area (μm2)
Average area of lipid droplet (μm2)
WT
aKO

Supplementary Fig. 3: Generation and morphological characteristics of adipocyte-specific *Ssu72* conditional knockout mice (*Ssu72*$^{f/f}$; *Adiponectin-Cre*, Ssu72 aKO). **a**, Schematic representation of *Ssu72* locus, targeting vector, and deleted (*floxed*) loci. Exons 1-2 and restriction sites (Bh, BamHI) are shown in the *Ssu72* locus. The targeting strategy was designed to flank *Ssu72* exon 1 with *loxP* sites, allowing Cre-mediated exon 1 deletion. **b**, Relative mRNA expression levels of *Ssu72* in eWAT, iWAT, and BAT from 6-week-old Ssu72 WT and aKO male mice (n = 5 mice per group). Data are presented as mean ± SEM. Statistical comparisons were made using two-tailed unpaired Student's t-test. **c**, Extracts of eWAT, iWAT, and BAT from 6-week-old Ssu72 WT and aKO male mice were harvested and immunoblotted with anti-Ssu72 and anti-GAPDH antibodies (n = 2 mice per group). **d**, Body weight gain in male WT and aKO mice fed normal chow diet (NCD) (n = 4 mice per group). Data are presented as mean ± SEM. Statistical comparisons were made using two-way ANOVA. **e**, Weights of BAT from 10-week-old Ssu72 WT and aKO male mice (n = 6 mice per group). Data are presented as mean ± SEM. Statistical comparisons were made using two-tailed unpaired Student's t-test (p = 0.3027). **f**, Representative images of iWAT and eWAT from 6-, and 22-week-old WT and aKO male mice. **g**,**h**, H&E staining of representative sections and size analyses of lipid droplets using ImageJ software from iWAT (**g**) and eWAT (**h**) of WT and aKO mice (scale bar, 100 μm; upper panels). The distribution plot of the lipid droplet area and the average area of lipid droplet in WT and aKO mice (n = 4 mice per group; bottom panels). Data are presented as mean ± SEM. Statistical comparisons were made using two-tailed unpaired Student's t-test. Source data are provided as a Source Data file.

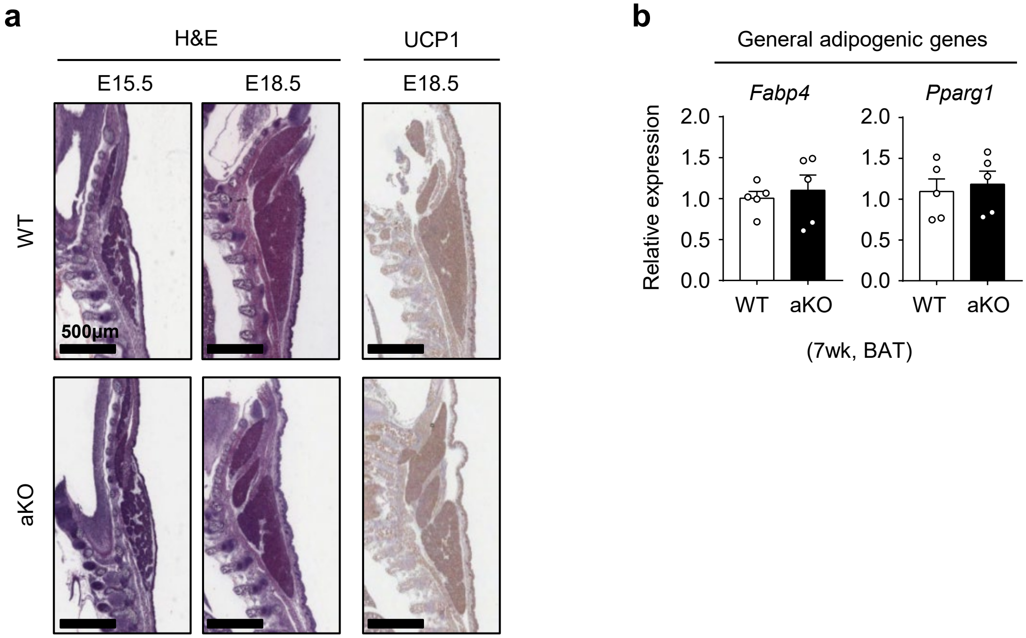

Supplementary Fig. 4: Adipose Ssu72 deficiency may not affect BAT development or adipogenesis. a, H&E staining and Ucp1 immunohistochemical staining of representative sections from Ssu72 WT and aKO mice embryos (E) at 15.5 and 18.5 days. **b**, Relative mRNA expression levels of general adipogenic genes (*Fabp4* and *Pparg1*) in BAT from 7-week-old WT and aKO male mice housed at RT (n = 5 mice per group). Data are presented as mean ± SEM. Statistical comparisons were made using two-tailed unpaired Student's t-test (*Fabp4*, p = 0.6486; *Pparg1*, p = 0.6962). Source data are provided as a Source Data file.

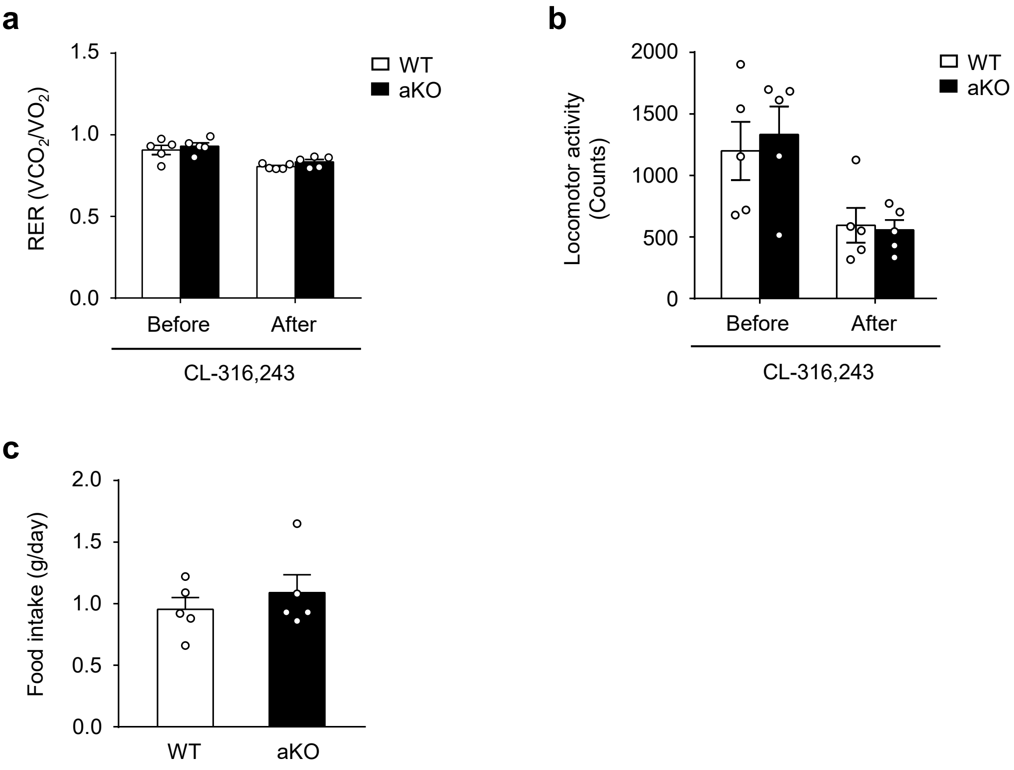

Supplementary Fig. 5: Ssu72 deficiency does not affect locomotor activity or daily food intake of mice. a,**b**, 7-week-old Ssu72 WT and aKO male mice were treated with 10mg/kg CL-316,243 (*n* = 5 mice per group). Respiratory exchange ratio (VCO_2/VO_2) of mice (**a**) and locomotor activities of mice before and after CL-316,243 treatment (**b**). Data are presented as mean ± SD. Statistical comparisons were made using two-way ANOVA. **c**, Daily food intake measurement in Ssu72 WT and aKO male mice (*n* = 5 mice per group). Data were calculated as average change in body weight by average food intake. Data are presented as mean ± SEM. Statistical comparisons were made using two-tailed unpaired Student's *t*-test. Source data are provided as a Source Data file.

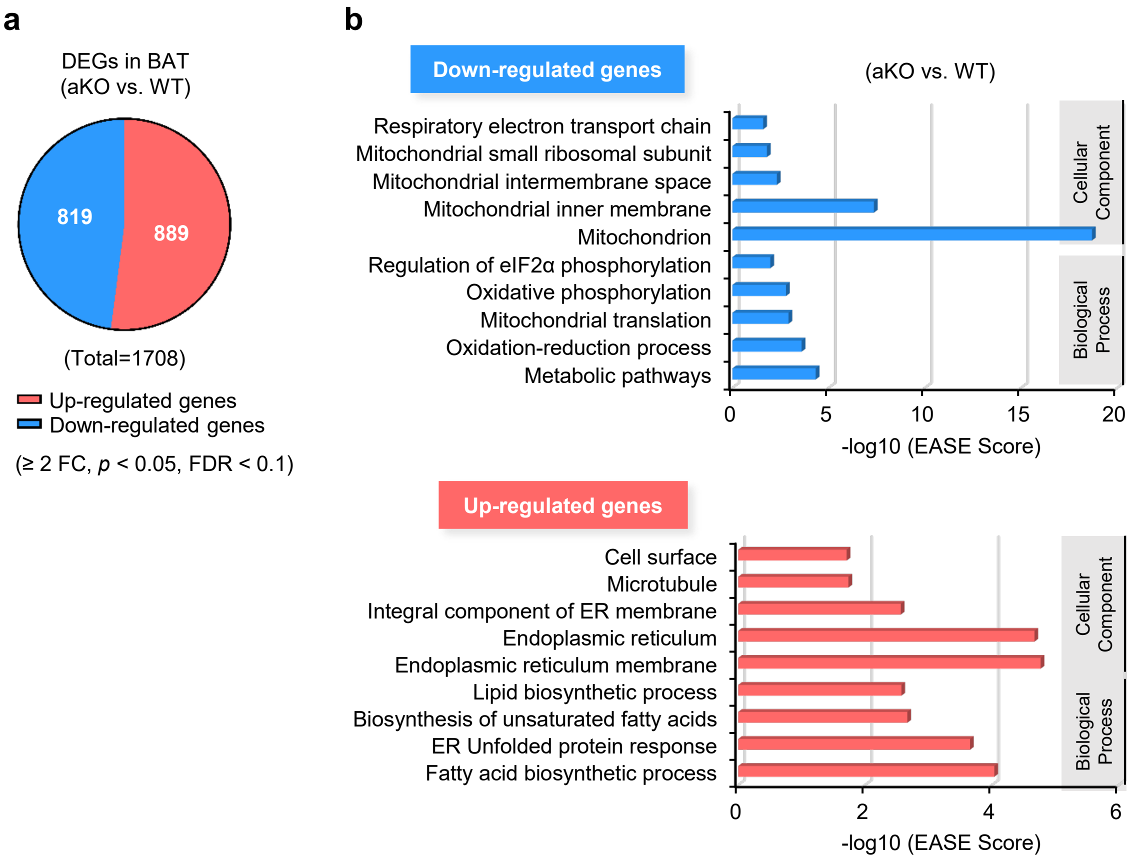

Supplementary Fig. 6: Transcriptional profiling of Ssu72-ablated BAT reveals impaired mitochondrial homeostasis and changes in regulation of fatty acid metabolism and unfolded protein response (UPR) signaling pathways. a, Pie chart indicating differentially expressed genes (DEGs) of aKO BAT compared to those of WT BAT based on false discovery rate (FDR)-adjusted $p < 0.05$ and fold change (FC) ≥ 2. Among 1,708 differentially expressed genes, 889 genes (red) were upregulated whereas the rest 819 genes (blue) were downregulated ($p < 0.05$, FDR < 0.1) Both 8-week-old Ssu72 WT ($n = 3$) and aKO male mice ($n = 3$) were housed at RT. *p* value was calculated as two-sided t-test. Exact *p* values of each statistical test are available in the processed RNA-seq data file (Supplementary Data 1). **b**, Gene ontology (GO) enrichment analysis of differentially expressed genes in BAT. GO biological process enrichment analyses of downregulated and upregulated genes in BAT from Ssu72 aKO mice relative to that from Ssu72 WT mice were performed. Source data are provided as a Source Data file.

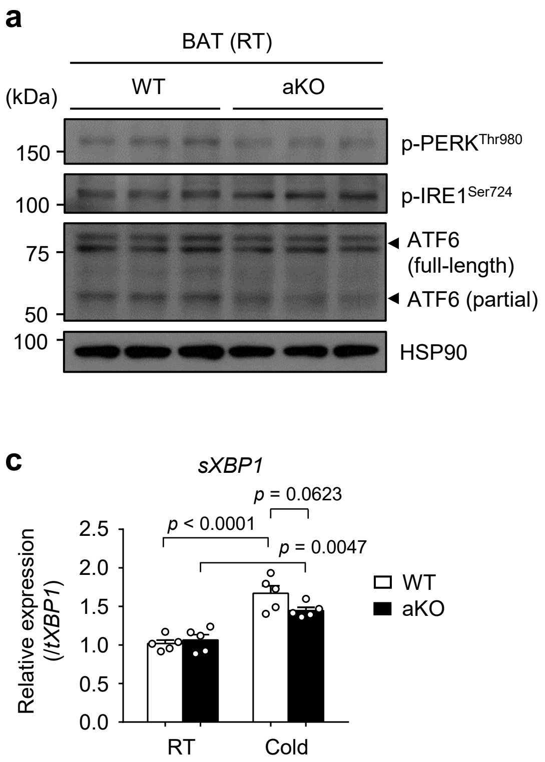

Supplementary Fig. 7: Ssu72 depletion in BAT induces eIF2α phosphorylation during cold adaptation without affecting IRE1α-Xbp1 pathway. a, Expression of activated three ER stress sensors of UPR^{ER} in BAT from 11-week-old Ssu72 WT and aKO male mice housed at RT (n = 3 mice per group). **b**, Western blots in lysates from BAT from 11-week-old Ssu72 WT and aKO male mice exposed to acute cold (4℃) for 4 hours (n = 3 mice per group). **c**, Relative mRNA expression levels of spliced *Xbp1* (*sXbp1*) (n = 5 mice per group). Total *Xbp1* (*tXBP1*) was used as a normalization control. Data are presented as mean ± SEM. Statistical comparisons were made using two-way ANOVA. Source data are provided as a Source Data file.

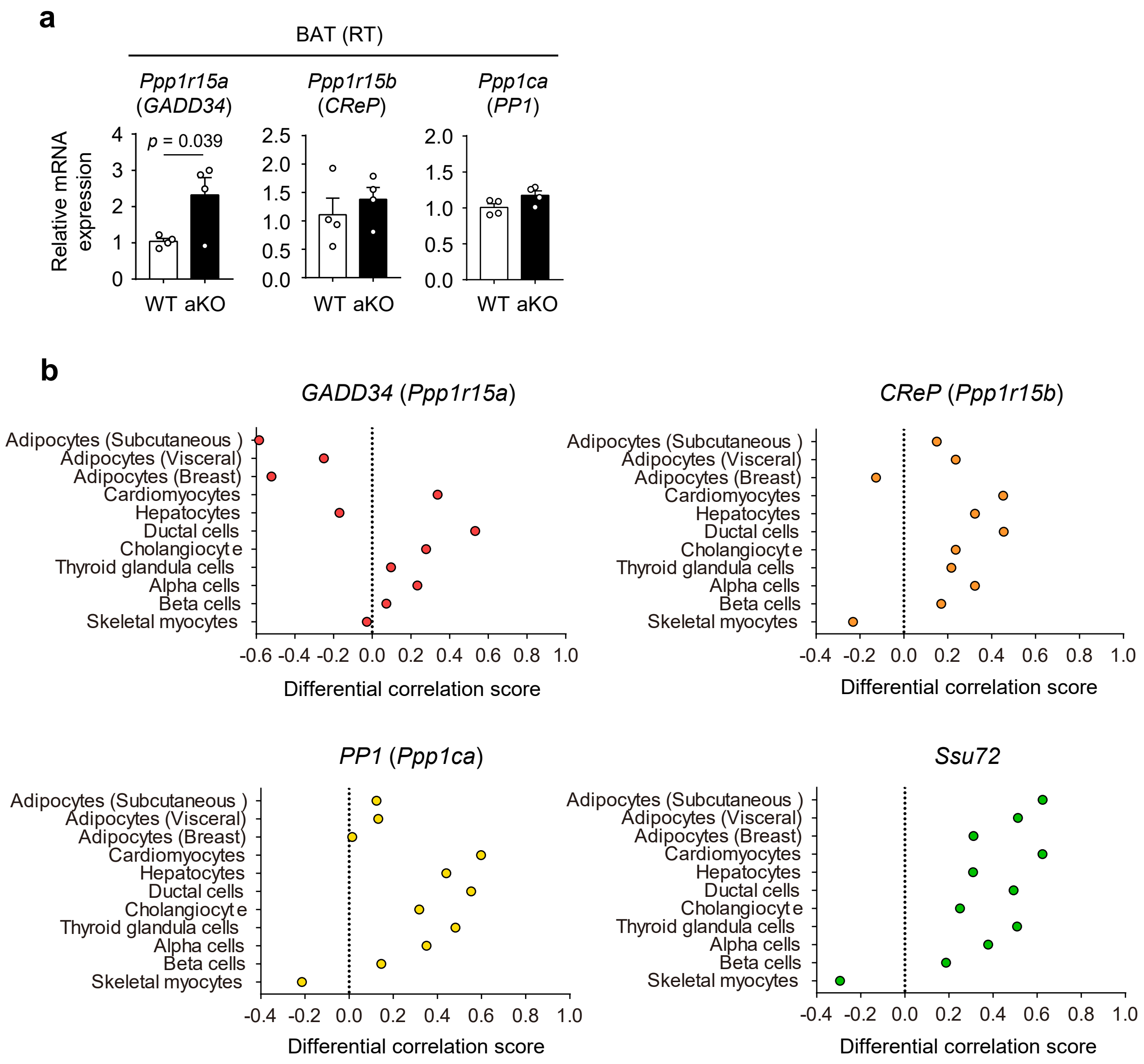

Supplementary Fig. 8: Other eIF2α phosphatases appear to have less effect on brown adipocytes in Ssu72 aKO mice. a, Relative mRNA expression levels of eIF2α phosphatases (*Ppp1r15a*, *Ppp1r15b*, *Ppp1ca*) in BAT of 11-week-old Ssu72 WT and aKO male mice housed at RT (23°C) (n = 4 mice per group). Data are presented as mean ± SEM. Statistical comparisons were made using two-tailed unpaired Student's t-test. **b**, The analysis performed on data from RNA-seq of unfractioned human tissue samples. The results of the data analysis were provided by Human Protein Atlas (https://www.proteinatlas.org/). Data is sourced from the Genotype-Tissue Expression (GTEx) portal (https://gtexportal.org/home/tissueSummaryPage). Reference transcripts with highly cell-type-specific expression were used for classification of each cell type[1]. The differential correlation score is the mean correlation between the specific gene and the reference transcripts. Source data are provided as a Source Data file.

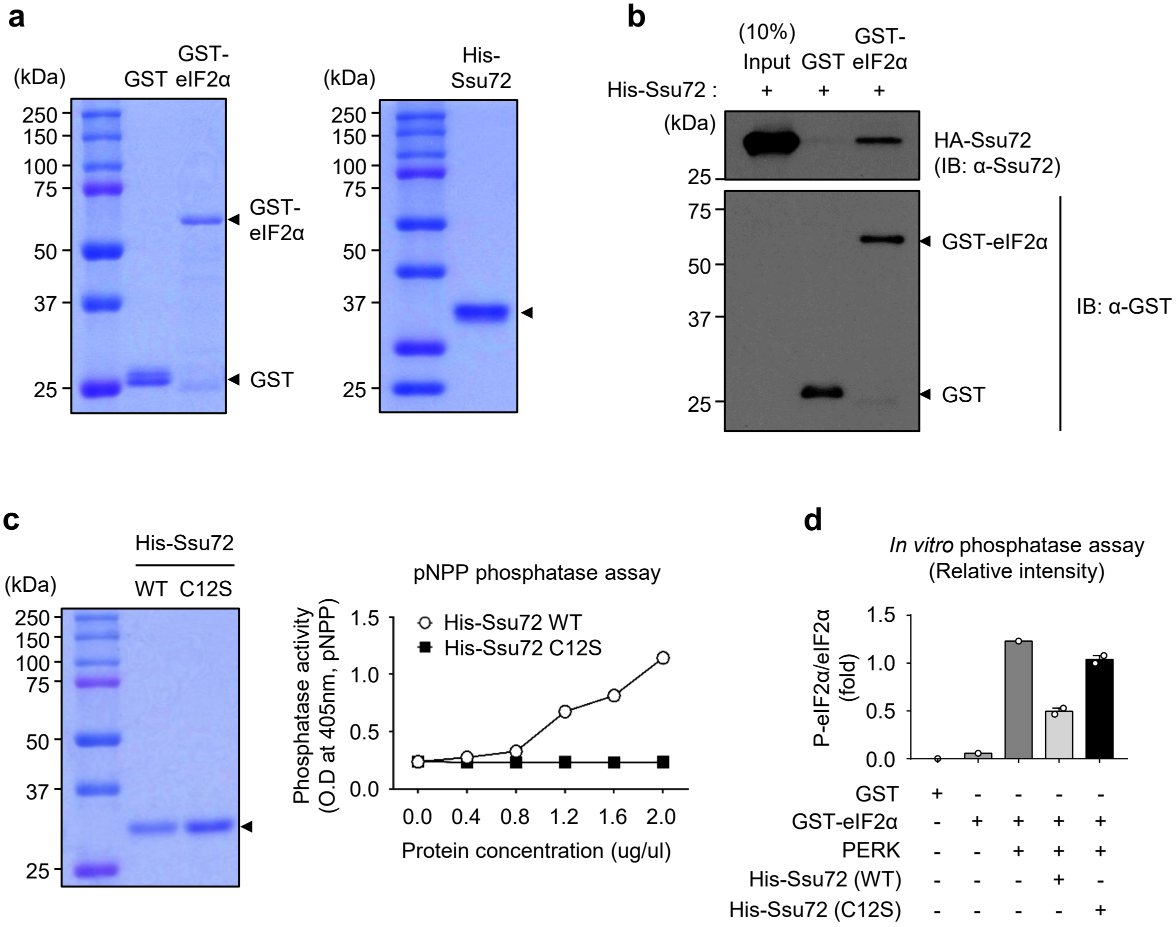

Supplementary Fig. 9: Ssu72 directly binds to eIF2α *in vitro*. **a**, Coomassie staining of purified glutathione S-transferase (GST) and GST-fused eIF2α (GST-eIF2α) protein (left), and His-fused Ssu72 (His-Ssu72) protein (right) ($n = 3$ independent biological replicates). **b**, GST pull-down assay with GST-eIF2α and His-Ssu72. GST and GST-eIF2α were bound to Glutathione Sepharose beads. Bead-bound GST and GST-eIF2α were incubated with indicated His-Ssu72 protein. Interaction of eIF2α and Ssu72 was analyzed by SDS-PAGE ($n = 3$ independent biological replicates). **c**, Purified His-fused Ssu72 (His-Ssu72) WT and phosphatase-inactive mutant (C12S) were reacted with p-nitrophenyl phosphate (pNPP) solution for phosphatase assay ($n = 3$ independent biological replicates). **d**, Quantification of phosphorylated GST-eIF2α levels normalized to GST-eIF2α levels shown in Fig. 4g using ImageJ (n = 1-2 per group). Data are presented as mean ± SEM. Source data are provided as a Source Data file.

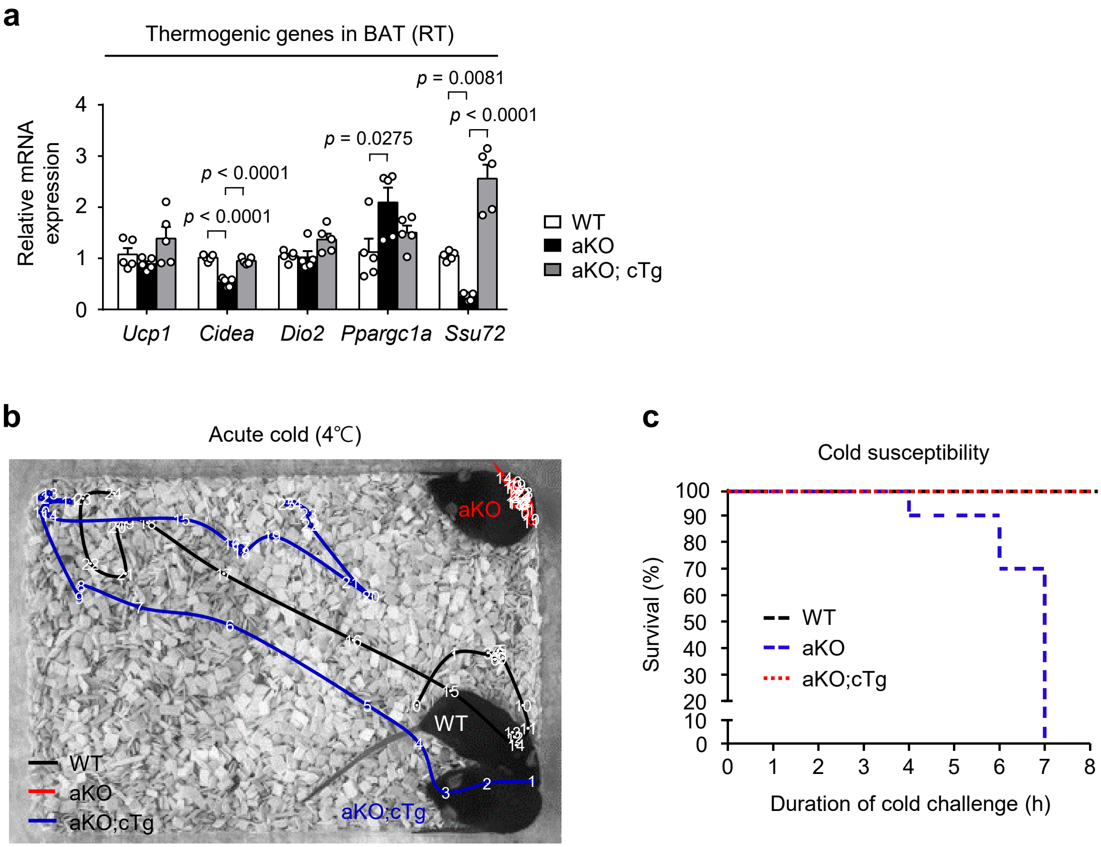

Supplementary Fig. 10: Cold intolerance in Ssu72 aKO mice is restored by ectopic Ssu72 expression in aKO;cTg mice. a, Relative mRNA expression levels of thermogenic genes (*Ucp1*, *Cidea*, *Dio2*, *Ppargc1α*) and *Ssu72* in BAT from 12-week-old Ssu72 WT and aKO female mice housed at RT (n = 5 mice per group). Data are presented as mean ± SEM. Statistical comparisons were made using one-way ANOVA. **b**, A representative trajectory plot of 7-week-old Ssu72 WT, aKO, aKO;cTg male mice with acute cold stress (4°C) (shown in Supplementary Video 1). After acute cold exposure (4°C) for 4 hours, distances travelled were simultaneously recorded at 25 frames/second for 1 minute. Each marked number represents the recording time (sec). **c**, Survival rate (%) of 7-week-old Ssu72 WT, aKO, and aKO;cTg male mice against cold exposure (4°C) (n = 10 mice per group). Source data are provided as a Source Data file.

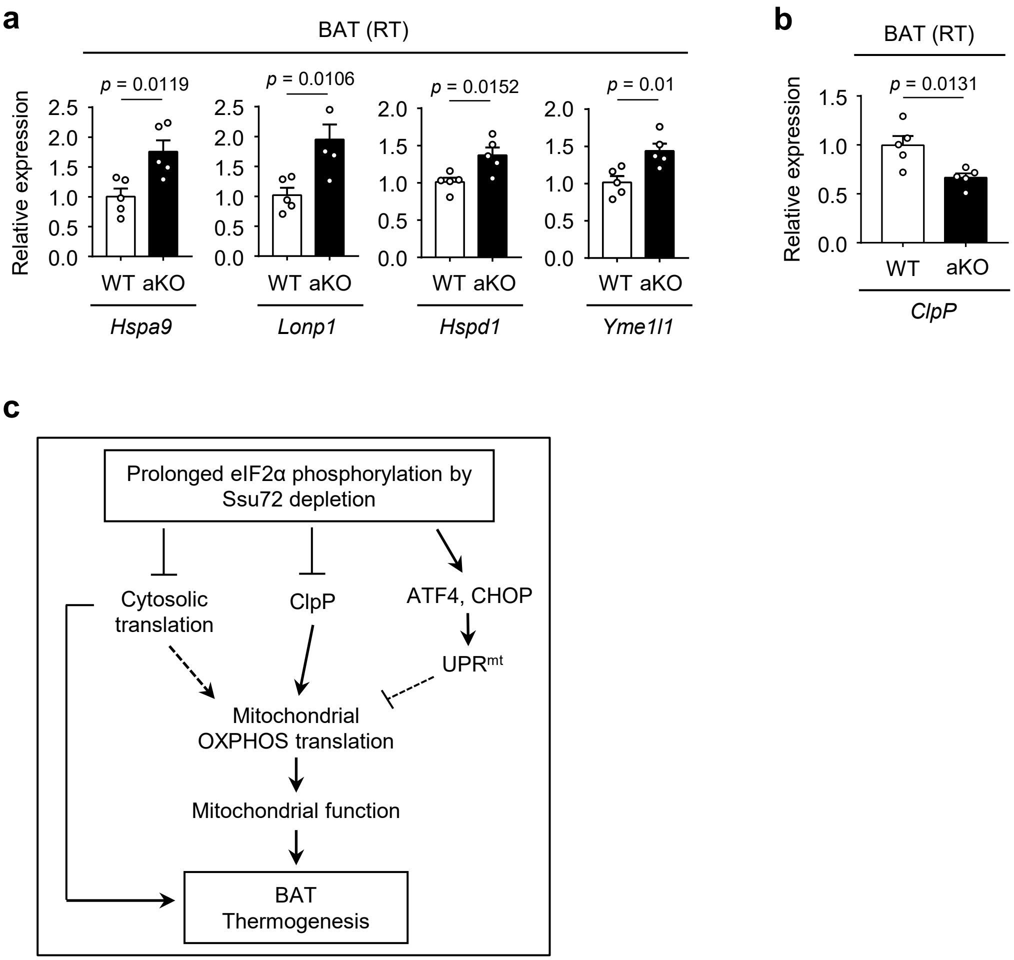

Supplementary Fig. 11: Prolonged eIF2α phosphorylation by Ssu72 depletion in BAT induces UPR^{mt} activation and suppresses ClpP expression. a,**b**, Relative mRNA expression levels of mitochondrial UPR (UPR^{mt}) genes (*Hspa9*, *Lonp1*, *Hspd1*, *Yme1l1*) (**a**) and *ClpP* genes (**b**) in BAT from 11-week-old Ssu72 WT and Ssu72 aKO male mice housed at RT ($n = 5$ mice per group). Data are presented as mean ± SEM. Statistical comparisons were made using Student's *t*-test. **c**, Diagrammatic representation of prolonged phosphorylation of eIF2α by Ssu72 deletion leading to global translational attenuation and mitochondrial dysfunction. Source data are provided as a Source Data file.

Supplementary Table 1: List of primer sequences for qRT-PCR[2-7].

qRT-PCR primer	Forward (5'-3')	Reverse (5'-3')
Ssu72	CATGGAAGCACACAACATCCTCA	TGGACGGGGCTTGATCCTTTTAT
Ucp1	ACTGCCACACCTCCAGTCATT	CTTTGCCTCACTCAGGATTGG
Ppargc1α	CCCTGCCATTGTTAAGACC	TGCTGCTGTTCCTGTTTTC
Elovl3	GATGGTTCTGGGCACCATCTT	CGTTGTTGTGTGGCATCCTT
Cidea	ATCACAACTGGCCTGGTTACG	TACTACCCGGTGTCCATTTCT
Cpt1b	GGTCCCATAAGAAACAAGACCTCC	CAGAAAGTACCTCAGCCAGGAAAG
Ppara	GGATGTCACACAATGCAATTCG	TCACAGAACGGCTTCCTCAGGT
Fabp4	GATGAAATCACCGCAGACGACA	ATTGTGGTCGACTTTCCATCCC
Ppar-γ	GAGTGTGACGACAAGATTTG	GGTGGGCCAGAATGGCATCT
mtDNA	TTAAGACACCTTGCCTAGCCACAC	CGGTGGCTGGCACGAAATT
NucDNA	ATGACGATATCGCTGCGCTG	TCACTTACCTGGTGCCTAGGGC
Atf3	AGCGAAGACTGGAGCAAAATG	TCCGATGGCAGAGGTGTTTA
Ddit3 (CHOP)	CATACACCACCACACCTGAAAG	CCGTTTCCTAGTTCTTCCTTGC
Fgf21	TCTCTATGGATCGCCTCACTT	CCATGGGCTTCAGACTGGTA
sXbp1	GCTGAGTCCGCAGCAGGT	CAGGGTCCAACTTGTCCAGAAT
tXbp1	TGAAAAACAGAGTAGCAGCGCAGA	CCCAAGCGTGTTCTTAACTC
Ppp1r15a	CTCTAAAAGCTCGGAAGGTACAC	GCTTCGATCTCGTGCAAACT
Ppp1r15b	CCAGCATGGGGAGTTTAGCA	AGGACCTTGCACATGTAGGC
Ppp1ca	AGCTCAACCTGGACTCCATC	GCCATGGATGTCACCACAG
Hspa9	GACAAGGATGCCCAAGGTTC	CTGTAACGTCACCAGCCAAC
Hspd1	AGTGTTCAGTCCATTGTCCC	TGACTGCCACAACCTGAAG
Lonp1	CTTCCGTTTCAGTGTTGGTG	GGGTTCTCTGTCTTGGTCTTC
ClpP	ATATACTCGAGGCTGTTGCG	CCACCTGGGCTGTTGATATAC
Ndufb8	ACATCTCTTCGGCTTTGTGG	CAGGCTCTTTGGTAGGATCAC
Sdhb	ACCCCTTCTCTGTCTACCG	AATGCTCGCTTCTCCTTGTAG
Uqcrc2	TTCCAGTGCAGATGTCCAAG	CTGTTGAAGGACGGTAGAAGG
Cox4i1	ATGTCACGATGCTGTCTGCC	GTGCCCCTGTTCATCTCGGC
Atp5a1	CATTGGTGATGGTATTGCGC	TCCCAAACACGACAACTCC
MT-ND1	ACGCTTCCGTTACGATCAAC	ACTCCCGCTGTAAAAATTGG
MT-ND2	TTTCATAGGGGCATGAGGAG	GTGAGGGATGGGTTGTAAGG
MT-CO1	GACACACGAGCTTACTTTAC	GCTATGATAGCAAACACTGC
MT-CO2	CCTCCACTCATGAGCAGTCC	GAATAACCCTGGTCGGTTTG
MT-CO3	TAACCCTTGGCCTACTCACC	ATAGGAGTGTGGTGGCCTTG
MT-ATP6	CAACCGTCTCCATTCTTTCC	TCATGTTCGTCCTTTTGGTG
MT-ATP8	GGCACCTTCACCAAAATCAC	TTGTTGGGGTAATGAATGAGG
18S rRNA	CGCCGCTAGAGGTGAAATTCT	CATTCTTGGCAAATGCTTTCG
5S rRNA	GGCCATACCACCCTGAAC	AGCCTACAGCACCCGGTAT

Supplementary Table 2: List of all antibodies used in this study.

Antibody	SOURCE	IDENTIFIER
Rabbit anti-Ssu72	Cell signaling Technology	Cat# 12816; RRID:AB_2798033
Rabbit anti-PP1α	Cell signaling Technology	Cat# 2582; RRID:AB_330822
Rabbit anti-CDC25B	Thermo Fisher Scientific	Cat# PA5-17759; RRID:AB_11003214
Rabbit anti-PTEN	Cell signaling Technology	Cat# 9188; RRID:AB_2253290
Mouse anti-PP2A-B56-α	Santa Cruz Biotechnology	Cat# sc-271311; RRID:AB_10610392
Rabbit anti-β-actin	Sigma-Aldrich	Cat# A2066; RRID:AB_476693
Goat anti-UCP1	Santa Cruz Biotechnology	Cat# sc-6529; RRID:AB_2213781
Rabbit anti-GAPDH	Cell signaling Technology	Cat# 2118; RRID:AB_561053
Rabbit anti-PGC1α	Santa Cruz Biotechnology	Cat# sc-13067; RRID:AB_2166218
Mouse anti-HSP90	Santa Cruz Biotechnology	Cat# sc-13119; RRID:AB_675659
Rabbit anti-phospho-eIF2α (Ser51)	Cell signaling Technology	Cat# 3398; RRID:AB_2096481
Rabbit anti-eIF2α	Cell signaling Technology	Cat# 5324; RRID:AB_10692650
Mouse anti-ATF4	Santa Cruz Biotechnology	Cat# sc-390063; RRID:AB_2810998
Mouse anti-CHOP	Cell signaling Technology	Cat# 2895; RRID:AB_2089254
Rabbit anti-phospho-PERK (Thr980)	Thermo Fisher Scientific	Cat# MA5-15033; RRID:AB_10980432
Rabbit anti-phospho-IRE1α (Ser724)	Thermo Fisher Scientific	Cat# PA1-16927; RRID:AB_2262241
Rabbit anti-ATF-6	Cell signaling Technology	Cat# 65880; RRID:AB_2799696
Mouse anti-Myc-Tag	Cell signaling Technology	Cat# 2276; RRID:AB_331783
Mouse anti-HA-Tag	Santa Cruz Biotechnology	Cat# sc-7392; RRID:AB_627809
Rabbit anti-HA-Tag	Cell signaling Technology	Cat# 3724; RRID:AB_1549585
Rabbit anti-β-actin	Cell signaling Technology	Cat# 3700; RRID:AB_2242334
Mouse anti-GST	Santa Cruz Biotechnology	Cat# sc-138; RRID:AB_627677
Mouse anti-puromycin	DSHB	Cat# PMY-2A4; RRID:AB_2619605
Rabbit anti-AMPKα	Cell signaling Technology	Cat# 5831; RRID:AB_10622186
Rabbit anti-PKA C alpha	GeneTex	Cat# GTX104934; RRID:AB_2037751
Rabbit anti-UCP1	Abcam	Cat# ab10983; RRID:AB_2241462
Mouse anti-ClpP	Santa Cruz Biotechnology	Cat# sc-271284; RRID:AB_10610081
Mouse anti-total OXPHOS Rodent WB Antibody Cocktail	Abcam	Cat# ab110413; RRID:AB_2629281
Rabbit anti-COX IV	Cell signaling Technology	Cat# 4850; RRID:AB_2085424
Rabbit anti-MT-ND1	Abcam	Cat# ab181848; RRID:AB_2687504
Goat anti-Rabbit IgG(H+L)-HRP	GenDEPOT	Cat# SA002-500
Goat anti-Mouse IgG(H+L)-HRP	GenDEPOT	Cat# SA001-500
Rabbit anti-Goat IgG(H+L)-HRP	GenDEPOT	Cat# SA007-500
Goat anti-Rabbit IgG (H+L) Secondary Antibody, Alexa Fluor 568	Thermo Fisher Scientific	Cat# A-11011; RRID:AB_143157
Biotinylated goat anti-rabbit IgG secondary antibody	Vector Laboratories	Cat# PK-6101, RRID:AB_2336820

Supplementary References

1. Norreen-Thorsen, M. et al. A human adipose tissue cell-type transcriptome atlas. Cell Rep 40, 111046 (2022).

2. Wall, C. E. et al. High-fat diet and FGF21 cooperatively promote aerobic thermogenesis in mtDNA mutator mice. Proc Natl Acad Sci U S A 112, 8714-8719 (2015).

3. Masand, R. et al. Proteome Imbalance of Mitochondrial Electron Transport Chain in Brown Adipocytes Leads to Metabolic Benefits. Cell Metab 27, 616-629 e614 (2018).

4. Choi, H. R. et al. Dual-specificity phosphatase 10 controls brown adipocyte differentiation by modulating the phosphorylation of p38 mitogen-activated protein kinase. PLoS One 8, e72340 (2013).

5. Wardelmann, K. et al. Insulin action in the brain regulates mitochondrial stress responses and reduces diet-induced weight gain. Mol Metab 21, 68-81 (2019).

6. Yoon, S. B. et al. Real-time PCR quantification of spliced X-box binding protein 1 (XBP1) using a universal primer method. PLoS One 14, e0219978 (2019).

7. Park, H. et al. Peroxisome-derived lipids regulate adipose thermogenesis by mediating cold-induced mitochondrial fission. J Clin Invest 129, 694-711 (2019).

Uncropped gels and blots for Supplementary Figures

In Supplementary Fig. 2

c

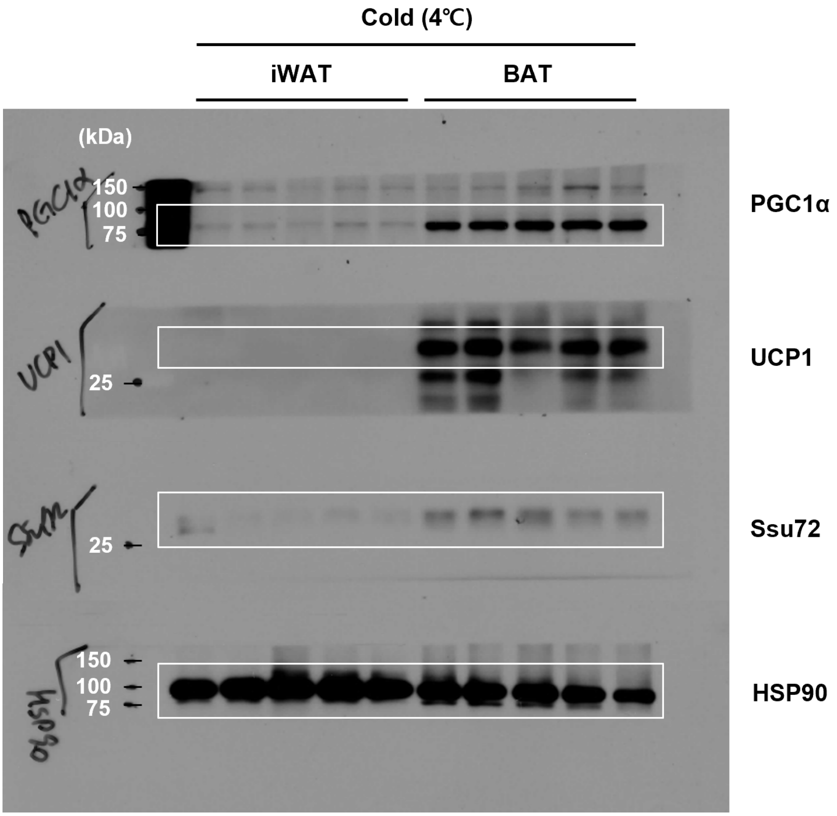

Uncropped gels and blots for Supplementary Figures

In Supplementary Fig. 2

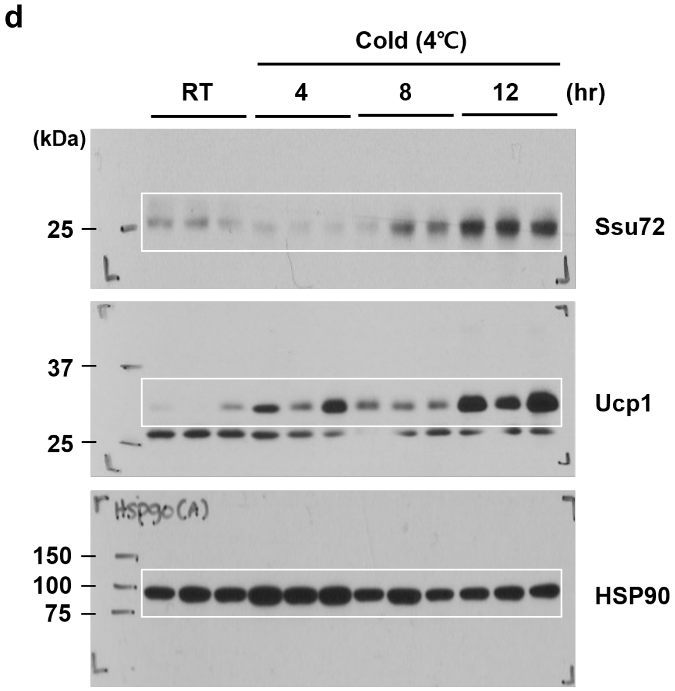

In Supplementary Fig. 3

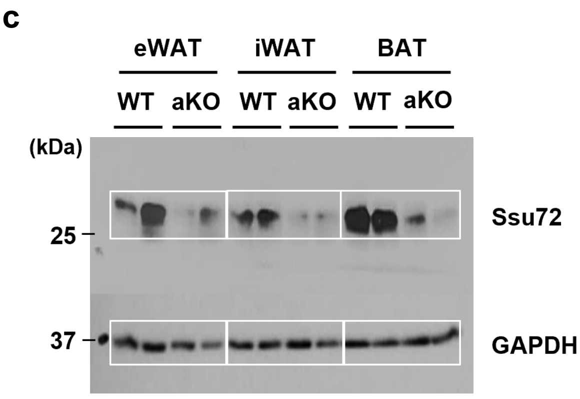

Uncropped gels and blots for Supplementary Figures

In Supplementary Fig. 7

a

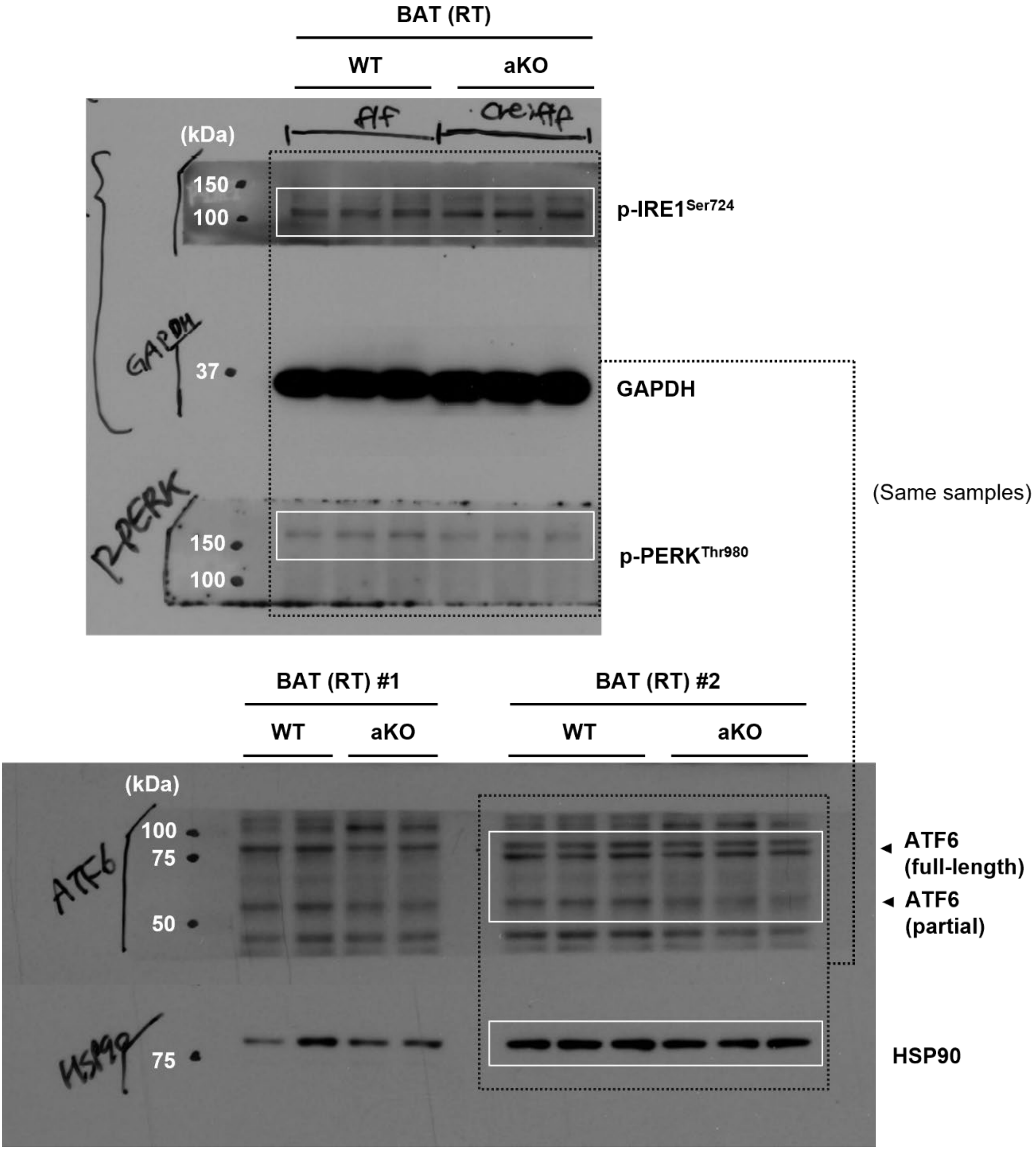

Uncropped gels and blots for Supplementary Figures

In Supplementary Fig. 7

b

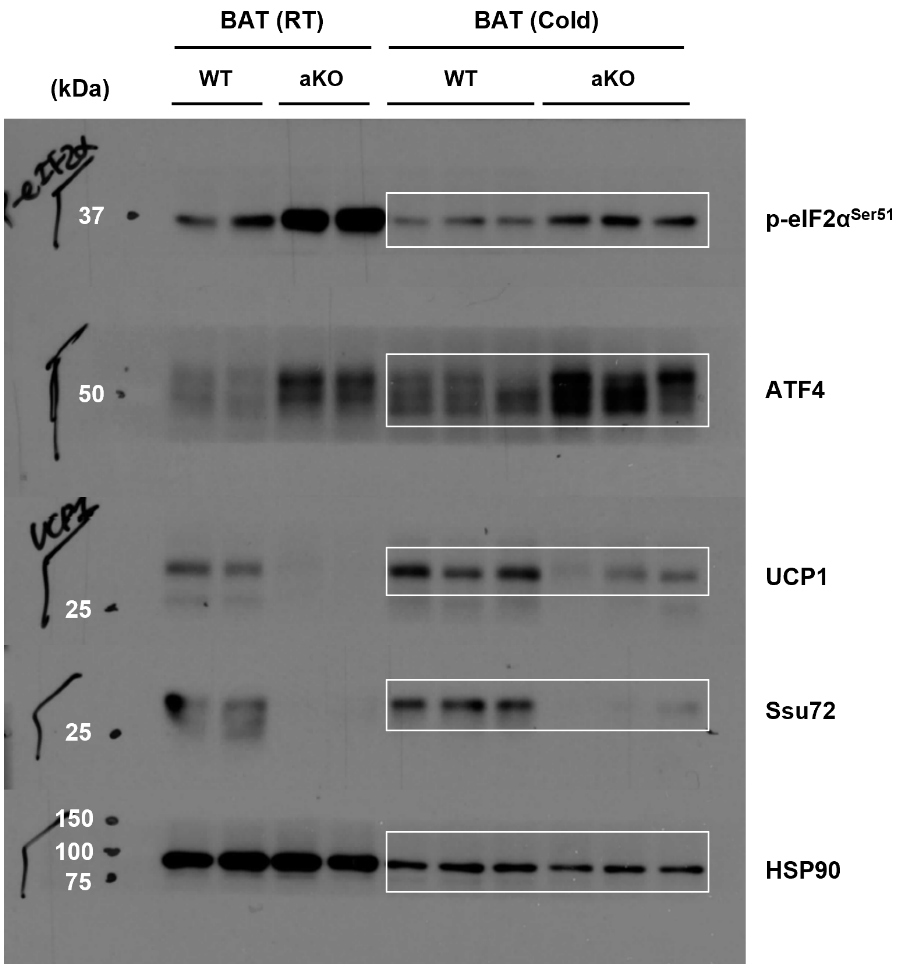

Uncropped gels and blots for Supplementary Figures

In Supplementary Fig. 9

a

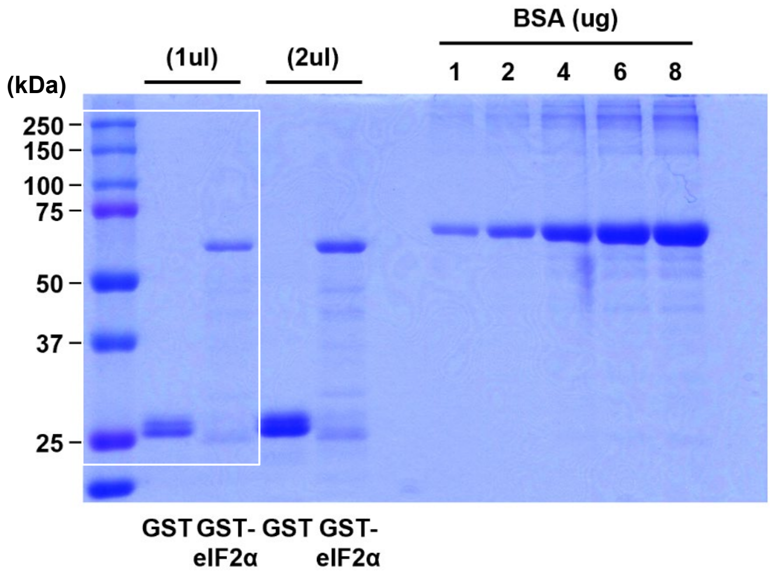

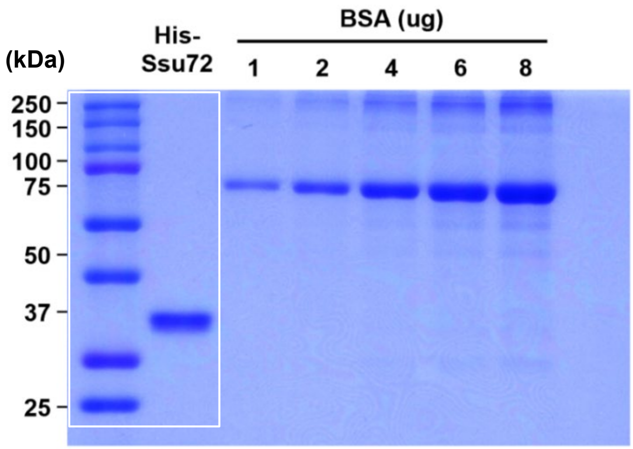

Uncropped gels and blots for Supplementary Figures

In Supplementary Fig. 9

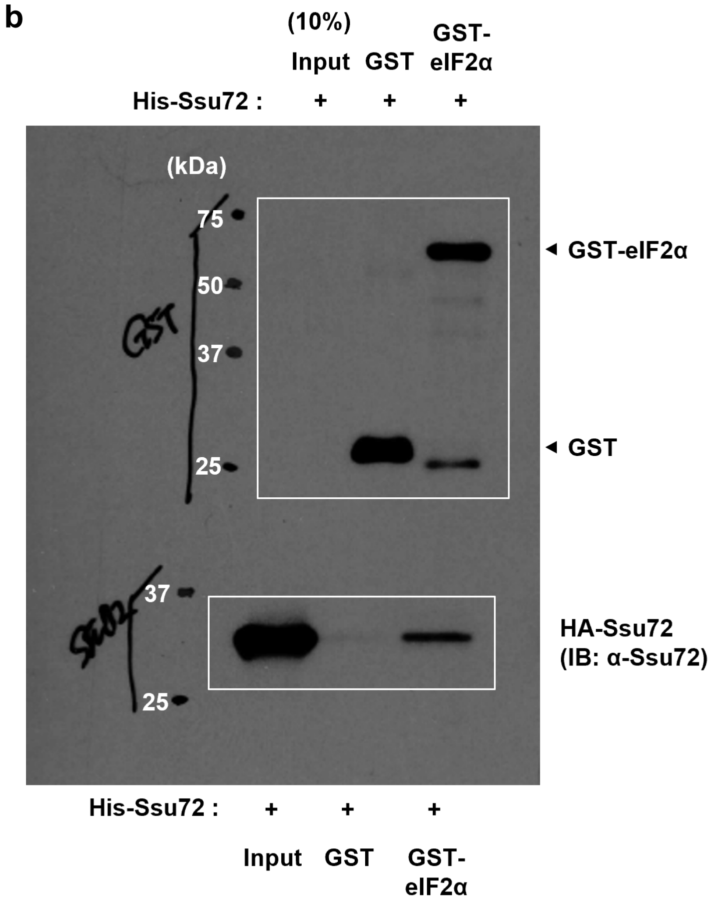

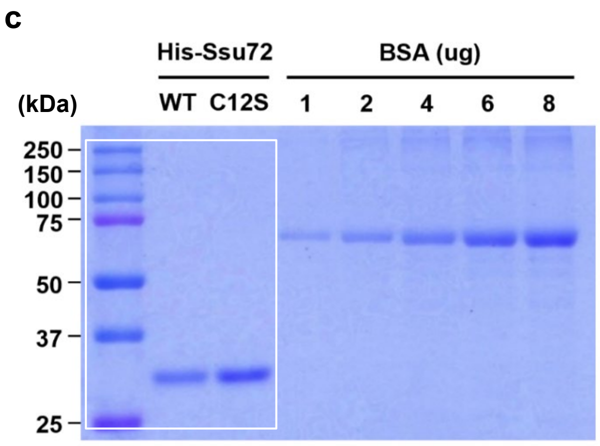

Supplement: Supplementary file 1 — Supplementary Information [file 41467_2023_36836_MOESM1_ESM.pdf]
