# Supplementary material for: Ssu72 phosphatase is essential for thermogenic adaptation by regulating cytosolic translation
Source: Nat Commun. 2023 Feb 25;14:1097. doi: 10.1038/s41467-023-36836-y (PMC9968297; doi:10.1038/s41467-023-36836-y)
Supplement: Supplementary file 3 — Description of Additional Supplementary Files [file 41467_2023_36836_MOESM3_ESM.pdf]

Description of Additional Supplementary Files

Supplementary Movie 1: Behaviors of Ssu72 WT (WT), aKO (KO) and aKO; cTg (Tg) mice against acute cold stress (4℃), related to Supplementary Fig. 10.

Supplementary Data 1: List of genes differentially expressed in BAT between Ssu72 WT and Ssu72 aKO mice.
