# Supplementary material for: Ssu72 phosphatase is essential for thermogenic adaptation by regulating cytosolic translation
Source: Nat Commun. 2023 Feb 25;14:1097. doi: 10.1038/s41467-023-36836-y (PMC9968297; doi:10.1038/s41467-023-36836-y)
Supplement: Supplementary file 6 — Reporting Summary [file 41467_2023_36836_MOESM6_ESM.pdf]

nature portfolio

Corresponding author(s): Hyun-Soo Kim, Min-Woo Lee, and Chang-Woo Lee

Last updated by author(s): Feb 3, 2023

Reporting Summary

Nature Portfolio wishes to improve the reproducibility of the work that we publish. This form provides structure for consistency and transparency in reporting. For further information on Nature Portfolio policies, see our Editorial Policies and the Editorial Policy Checklist.

Statistics

For all statistical analyses, confirm that the following items are present in the figure legend, table legend, main text, or Methods section.

n/a	Confirmed	
☐	☒	The exact sample size (*n*) for each experimental group/condition, given as a discrete number and unit of measurement
☐	☒	A statement on whether measurements were taken from distinct samples or whether the same sample was measured repeatedly
☐	☒	The statistical test(s) used AND whether they are one- or two-sided<br>*Only common tests should be described solely by name; describe more complex techniques in the Methods section.*
☐	☒	A description of all covariates tested
☐	☒	A description of any assumptions or corrections, such as tests of normality and adjustment for multiple comparisons
☐	☒	A full description of the statistical parameters including central tendency (e.g. means) or other basic estimates (e.g. regression coefficient) AND variation (e.g. standard deviation) or associated estimates of uncertainty (e.g. confidence intervals)
☐	☒	For null hypothesis testing, the test statistic (e.g. *F*, *t*, *r*) with confidence intervals, effect sizes, degrees of freedom and *P* value noted<br>*Give P values as exact values whenever suitable.*
☒	☐	For Bayesian analysis, information on the choice of priors and Markov chain Monte Carlo settings
☒	☐	For hierarchical and complex designs, identification of the appropriate level for tests and full reporting of outcomes
☒	☐	Estimates of effect sizes (e.g. Cohen's *d*, Pearson's *r*), indicating how they were calculated

Our web collection on statistics for biologists contains articles on many of the points above.

Software and code

Policy information about availability of computer code

Data collection	QuantStudio 6 Flex Real-Time PCR (Life technologies)<br>Hitachi H600AB transmission electron microscope at 75 kV<br>Axio Imager microscope (ZEISS)<br>FastQC (version 0.11.9)<br>Tophat (version 2.0.13)<br>MoticEasyScan Pro 6 (Motic)<br>Aperio ImageScope (version 12.4.3.5008) (https://www.leicabiosystems.com/digital-pathology/manage/aperio-imagescope/)
Data analysis	RNA-seq: Differential gene expression analysis was performed using cuffdiff package (version 2.2.0). Raw read counts were used and modeled based on a negative binomial distribution. Data were obtained from three independent experiments and processed using DAVID Bioinformatics Resources 6.7 software for Gene ontology, BIOCARTA, KEGG pathway, and gene set enrichment analyses.<br>Gene set enrichment analysis (GSEA): Software version 4.1.0 (Broad Institute and University of California, https://www.gsea-msigdb.org/gsea/index.jsp)<br>ImageJ (version 1.52a) (NIH, https://imagej.nih.gov/ij/)<br>GraphPad Prism software (version 7) (https://www.graphpad.com/scientific-software/prism/)

For manuscripts utilizing custom algorithms or software that are central to the research but not yet described in published literature, software must be made available to editors and reviewers. We strongly encourage code deposition in a community repository (e.g. GitHub). See the Nature Portfolio guidelines for submitting code & software for further information.

Data

Policy information about availability of data

All manuscripts must include a data availability statement. This statement should provide the following information, where applicable:

- Accession codes, unique identifiers, or web links for publicly available datasets
- A description of any restrictions on data availability
- For clinical datasets or third party data, please ensure that the statement adheres to our policy

Human transcriptomic datasets: accession number GSE2508
The processed RNA-seq data generated in this study are available in the Supplementary Data 1 and also available at figshare (https://doi.org/10.6084/m9.figshare.21971576.v2).
Raw data for all figures are provided in the Source Data file.
The molecular signature databases (MSigDB) for GSEA are provided on the GSEA MSigDB website (https://www.gsea-msigdb.org/gsea/msigdb/index.jsp).

Human research participants

Policy information about studies involving human research participants and Sex and Gender in Research.

Reporting on sex and gender	N/A
Population characteristics	N/A
Recruitment	N/A
Ethics oversight	N/A

Note that full information on the approval of the study protocol must also be provided in the manuscript.

Field-specific reporting

Please select the one below that is the best fit for your research. If you are not sure, read the appropriate sections before making your selection.

[x] Life sciences [ ] Behavioural & social sciences [ ] Ecological, evolutionary & environmental sciences

For a reference copy of the document with all sections, see nature.com/documents/nr-reporting-summary-flat.pdf

Life sciences study design

All studies must disclose on these points even when the disclosure is negative.

Sample size	Preliminary experiments were performed to estimate the variance for each assay and determine a sufficient sample size. Sample sizes are shown in the figure or stated in the figure legend.
Data exclusions	No data were excluded from analysis.
Replication	Animal experiments were performed with sufficient sample size, and n numbers for each experiment are stated in figure legends. All experiments were repeated at least three times and all attempts to replicate experiments were successful.
Randomization	For in vivo mice experiments, both age and sex-matched mice were grouped randomly according to the genotype and littermates were used where applicable. For in vitro experiments, cells are coming from the same populations and well location for cell culture were randomly arranged.
Blinding	Blinding was used for experiments involving pathological interpretation of histological sections and quantification of histological sections. Blinding was not used for any other experiments in this study since there was no need to avoid selection bias.

Reporting for specific materials, systems and methods

We require information from authors about some types of materials, experimental systems and methods used in many studies. Here, indicate whether each material, system or method listed is relevant to your study. If you are not sure if a list item applies to your research, read the appropriate section before selecting a response.

Materials & experimental systems

n/a	Involved in the study
	☒ Antibodies
	☒ Eukaryotic cell lines
☒	Palaeontology and archaeology
	☒ Animals and other organisms
☒	Clinical data
☒	Dual use research of concern

Methods

n/a	Involved in the study
☒	ChIP-seq
☒	Flow cytometry
☒	MRI-based neuroimaging

Antibodies

Antibodies used

Rabbit anti-Ssu72 (Cell signaling Technology, Cat# 12816), 1:2000
Rabbit anti-PP1α (Cell signaling Technology, Cat# 2582), 1:750
Rabbit anti-CDC25B (Thermo Fisher Scientific, Cat# PA5-17759), 1:1000
Rabbit anti-PTEN (Cell signaling Technology, Cat# 9188), 1:1000
Mouse anti-PP2A-B56-α (Santa Cruz Biotechnology, Cat# sc-271311), 1:1000
Rabbit anti-β-actin (Sigma-Aldrich, Cat# A2066), 1:3000
Goat anti-UCP1 (Santa Cruz Biotechnology, Cat# sc-6529), 1:750
Rabbit anti-GAPDH (Cell signaling Technology, Cat# 2118), 1:3000
Rabbit anti-PGC1α (Santa Cruz Biotechnology, Cat# sc-13067), 1:750
Mouse anti-HSP90 (Santa Cruz Biotechnology, Cat# sc-13119), 1:1000
Rabbit anti-phospho-eIF2α (Ser51) (Cell signaling Technology, Cat# 3398), 1:1000
Rabbit anti-eIF2α (Cell signaling Technology, Cat# 5324), 1:2000
Mouse anti-ATF4 (Santa Cruz Biotechnology, Cat# sc-390063), 1:1000
Mouse anti-CHOP (Cell signaling Technology, Cat# 2895), 1:750
Rabbit anti-phospho-PERK (Thr980) (Thermo Fisher Scientific, Cat# MA5-15033), 1:1000
Rabbit anti-phospho-IRE1α (Ser724) (Thermo Fisher Scientific, Cat# PA1-16927), 1:1000
Rabbit anti-ATF-6 (Cell signaling Technology, Cat# 65880), 1:1000
Mouse anti-Myc-Tag (Cell signaling Technology, Cat# 2276), 1:1500
Mouse anti-HA-Tag (Santa Cruz Biotechnology, Cat# sc-7392), 1:1000
Rabbit anti-HA-Tag (Cell signaling Technology, Cat# 3724), 1:2000
Mouse anti-β-actin (Cell signaling Technology, Cat# 3700), 1:3000
Mouse anti-GST (Santa Cruz Biotechnology, Cat# sc-138), 1:2000
Mouse anti-puromycin (DSHB, Cat# PMY-2A4), 1:200
Rabbit anti-AMPKα (Cell signaling Technology, Cat# 5831), 1:2000
Rabbit anti-PKA C alpha (GeneTex, Cat# GTX104934), 1:1000
Rabbit anti-UCP1 (Abcam, Cat#ab10983), 1:500
Mouse anti-ClpP (Santa Cruz Biotechnology, Cat# sc-271284), 1:1000
Mouse anti-total OXPHOS Rodent WB Antibody Cocktail (Abcam, Cat# ab110413), 1:500
Rabbit anti-COX IV (Cell signaling Technology, Cat# 4850), 1:1000
Rabbit anti-MT-ND1 (Abcam, Cat# ab181848), 1:1000
Goat anti-Rabbit IgG(H+L)-HRP (GenDEPOT, Cat# SA002-500), 1:7000
Goat anti-Mouse IgG(H+L)-HRP (GenDEPOT, Cat# SA001-500), 1:7000
Goat anti-Goat IgG(H+L)-HRP (GenDEPOT, Cat# SA007-500), 1:7000
Goat anti-Rabbit IgG (H+L) Secondary Antibody, Alexa Fluor 568 (Thermo Fisher Scientific, Cat# A-11011), 1:300
Biotinylated goat anti-rabbit IgG secondary antibody (Vector Laboratories, #PK-6101), 1:200

Validation

All antibodies used in this study are commercial (please see details above) and have been previously validated by the manufacturer:

Rabbit anti-Ssu72 (Cell signaling Technology, Cat# 12816) is validated for Western blot analysis and immunoprecipitation; this antibody works for human, mouse, rat, and monkey (https://www.cellsignal.com/products/primary-antibodies/ssu72-d3i2d-rabbit-mab/12816).
Rabbit anti-PP1α (Cell signaling Technology, Cat# 2582) is validated for Western blot analysis; this antibody works for human, mouse, rat, monkey, and D. melanogaster (https://www.cellsignal.com/products/primary-antibodies/pp1a-antibody/2582).
Rabbit anti-CDC25B (Thermo Fisher Scientific, Cat# PA5-17759) is validated for Western blot analysis and immunocytochemistry; this antibody works for human, mouse, rat, and non-human primate (https://www.thermofisher.com/antibody/product/Cdc25B-Antibody-Polyclonal/PA5-17759).
Rabbit anti-PTEN (Cell signaling Technology, Cat# 9188) is validated for Western blot analysis, immunoprecipitation, and immunohistochemical analysis; this antibody works for human, mouse, rat, monkey, and dog (https://www.cellsignal.com/products/primary-antibodies/pten-d4-3-xp-rabbit-mab/9188).
Mouse anti-PP2A-B56-α (Santa Cruz Biotechnology, Cat# sc-271311) is validated for Western blot analysis, immunoprecipitation, immunofluorescence analysis, and ELISA; this antibody works for human, mouse, and rat (https://www.scbt.com/p/pp2a-b56-alpha-antibody-f-4).
Rabbit anti-β-actin (Sigma-Aldrich, Cat# A2066) is validated for Western blot analysis, immunocytochemistry, and immunofluorescence analysis; this antibody works for wide range, vertebrates, human, slime mold, amoeba, chicken (https://www.sigmaaldrich.com/KR/ko/product/sigma/a2066).

Goat anti-UCP1 (Santa Cruz Biotechnology, Cat# sc-6529) is validated for Western blot analysis and immunofluorescence analysis; this antibody works for human, mouse, and rat (https://www.scbt.com/p/ucp1-antibody-m-17).
Rabbit anti-GAPDH (Cell signaling Technology, Cat# 2118) is validated for Western blot analysis, immunofluorescence analysis, flow cytometric analysis, and immunohistochemical analysis; this antibody works for human, mouse, rat, monkey, bovine, and pig (https://www.cellsignal.com/products/primary-antibodies/gapdh-14c10-rabbit-mab/2118).
Rabbit anti-PGC1α (Santa Cruz Biotechnology, Cat# sc-13067) is validated for Western blot analysis, immunoprecipitation, immunofluorescence analysis, and ELISA; this antibody works for human, mouse, and rat (https://www.scbt.com/p/pgc-1alpha-antibody-h-300).
Mouse anti-HSP90 (Santa Cruz Biotechnology, Cat# sc-13119) is validated for Western blot analysis, immunoprecipitation, immunofluorescence analysis, immunohistochemical analysis, and ELISA; this antibody works for human, mouse, and rat (https://www.scbt.com/p/hsp-90alpha-beta-antibody-f-8).
Rabbit anti-phospho-eIF2α (Ser51) (Cell signaling Technology, Cat# 3398) is validated for Western blot analysis, immunoprecipitation, and immunohistochemical analysis; this antibody works for human, mouse, rat, monkey, and D. melanogaster (https://www.cellsignal.com/products/primary-antibodies/phospho-eif2a-ser51-d9g8-xp-rabbit-mab/3398).
Rabbit anti-eIF2α (Cell signaling Technology, Cat# 5324) is validated for Western blot analysis, immunoprecipitation, and immunohistochemical analysis; this antibody works for human, mouse, rat, and monkey (https://www.cellsignal.com/products/primary-antibodies/eif2a-d7d3-xp-rabbit-mab/5324).
Mouse anti-ATF4 (Santa Cruz Biotechnology, Cat# sc-390063) is validated for Western blot analysis, immunoprecipitation, immunofluorescence analysis, immunohistochemical analysis, and ELISA; this antibody works for human, mouse, and rat (https://www.scbt.com/p/atf-4-antibody-b-3).
Mouse anti-CHOP (Cell signaling Technology, Cat# 2895) is validated for Western blot analysis, immunoprecipitation, immunofluorescence analysis, flow cytometric analysis, and chromatin immunoprecipitation; this antibody works for human, mouse, and rat (https://www.cellsignal.com/products/primary-antibodies/chop-l63f7-mouse-mab/2895).
Rabbit anti-phospho-PERK (Thr980) (Thermo Fisher Scientific, Cat# MA5-15033) is validated for Western blot analysis and immunohistochemistry; this antibody works for mouse and rat (https://www.thermofisher.com/antibody/product/Phospho-PERK-Thr980-Antibody-clone-G-305-4-Monoclonal/MA5-15033).
Rabbit anti-phospho-IRE1α (Ser724) (Thermo Fisher Scientific, Cat# PA1-16927) is validated for Western blot analysis, immunohistochemistry, immunocytochemistry, ELISA, immunoprecipitation, and chromatin immunoprecipitation; this antibody works for goat, human, mouse, non-human primate, pig, rabbit, and rat (https://www.thermofisher.com/antibody/product/Phospho-IRE1-alpha-Ser724-Antibody-Polyclonal/PA1-16927).
Rabbit anti-ATF-6 (Cell signaling Technology, Cat# 65880) is validated for Western blot analysis and immunoprecipitation; this antibody works for human and mouse (https://www.cellsignal.com/products/primary-antibodies/atf-6-d4z8v-rabbit-mab/65880).
Mouse anti-Myc-Tag (Cell signaling Technology, Cat# 2276) is validated for Western blot analysis, immunoprecipitation, Immunohistochemical analysis, immunofluorescence analysis, flow cytometric analysis, and chromatin immunoprecipitation; this antibody works for cells containing Myc-tagged protein (https://www.cellsignal.com/products/primary-antibodies/myc-tag-9b11-mouse-mab/2276).
Mouse anti-HA-Tag (Santa Cruz Biotechnology, Cat# sc-7392) is validated for Western blot analysis, immunoprecipitation, immunofluorescence analysis, and ELISA; this antibody works for cells containing HA-tagged protein (https://www.scbt.com/p/ha-probe-antibody-f-7).
Rabbit anti-HA-Tag (Cell signaling Technology, Cat# 3724) is validated for Western blot analysis, immunoprecipitation, immunohistochemical analysis, immunofluorescence analysis, flow cytometric analysis, and chromatin immunoprecipitation; this antibody works for cells containing HA-tagged protein (https://www.cellsignal.com/products/primary-antibodies/ha-tag-c29f4-rabbit-mab/3724).
Mouse anti-β-actin (Cell signaling Technology, Cat# 3700) is validated for Western blot analysis, immunohistochemical analysis, immunofluorescence analysis, and flow cytometric analysis; this antibody works for human, mouse, rat, hamster, monkey, and dog (https://www.cellsignal.com/products/primary-antibodies/b-actin-8h10d10-mouse-mab/3700).
Mouse anti-GST (Santa Cruz Biotechnology, Cat# sc-138) is validated for Western blot analysis and immunoprecipitation; this antibody works for GST fusion proteins of Schistosoma japonicum origin and recombinant GST fusion proteins expressed in E. coli (specifically designed to be used in combination with GST expression vectors such as pGEX.3X and pGEX.2T) (https://www.scbt.com/p/gst-antibody-b-14).
Mouse anti-puromycin (DSHB, Cat# PMY-2A4) is validated for Western blot analysis, immunohistochemical analysis, immunofluorescence analysis, and ELISA; this antibody works for all cells containing puromycin (https://dshb.biology.uiowa.edu/PMY-2A4).
Rabbit anti-AMPKα (Cell signaling Technology, Cat# 5831) is validated for Western blot analysis and immunoprecipitation; this antibody works for human, mouse, rat, monkey, and bovine (https://www.cellsignal.com/products/primary-antibodies/ampka-d5a2-rabbit-mab/5831).
Rabbit anti-PKA C alpha (GeneTex, Cat# GTX104934) is validated for Western blot analysis, immunofluorescence analysis, and immunohistochemical analysis; this antibody works for human and mouse (https://www.genetex.com/Product/Detail/PKA-C-alpha-antibody-N2C3/GTX104934).
Rabbit anti-UCP1 (Abcam, Cat#ab10983) is validated for Western blot analysis and immunohistochemical analysis; this antibody works for mouse and rat (https://www.abcam.com/ucp1-antibody-ab10983.html).
Mouse anti-ClpP (Santa Cruz Biotechnology, Cat# sc-271284) is validated for Western blot analysis, immunohistochemical analysis, immunofluorescence analysis, and ELISA; this antibody works for human, mouse, and rat (https://www.scbt.com/p/clpp-antibody-b-12).
Mouse anti-total OXPHOS Rodent WB Antibody Cocktail (Abcam, Cat# ab110413) is validated for Western blot analysis; this antibody works for human, mouse, rat, cow, and cynomolgus monkey (https://www.abcam.com/total-oxphos-rodent-wb-antibody-cocktail-ab110413.html).
Rabbit anti-COX IV (Cell signaling Technology, Cat# 4850) is validated for Western blot analysis, immunoprecipitation, immunohistochemical analysis, immunofluorescence analysis, flow cytometric analysis; this antibody works for human, mouse, rat, monkey, Zebrafish, bovine, and pig (https://www.cellsignal.com/products/primary-antibodies/cox-iv-3e11-rabbit-mab/4850).
Rabbit anti-MT-ND1 (Abcam, Cat# ab181848) is validated for Western blot analysis; this antibody works for human, mouse, and rat (https://www.abcam.com/mt-nd1-antibody-epr134662-ab181848.html).
Goat anti-Rabbit IgG(H+L)-HRP (GenDEPOT, Cat# SA002-500) is validated for Western blot analysis, immunohistochemical analysis,

immunofluorescence analysis, and ELISA; this antibody works rabbit (http://www.gendepot.com/product/list.php?item_search_type=all&item_search=SA002-500).
Goat anti-Mouse IgG(H+L)-HRP (GenDEPOT, Cat# SA001-500) is validated for Western blot analysis, immunohistochemical analysis, immunofluorescence analysis, and ELISA; this antibody works mouse (http://www.gendepot.com/product/list.php?item_search_type=all&item_search=SA001-500).
Goat anti-Goat IgG(H+L)-HRP (GenDEPOT, Cat# SA007-500) is validated for Western blot analysis, immunohistochemical analysis, immunofluorescence analysis, and ELISA; this antibody works goat (http://www.gendepot.com/product/list.php?item_search_type=all&item_search=SA007-500).
Goat anti-Rabbit IgG (H+L) Secondary Antibody, Alexa Fluor 568 (Thermo Fisher Scientific, Cat# A-11011) is validated for immunohistochemical analysis, immunofluorescence analysis, flow cytometric analysis; this antibody works rabbit (https://www.thermofisher.com/antibody/product/Goat-anti-Rabbit-IgG-H-L-Cross-Adsorbed-Secondary-Antibody-Polyclonal/A-11011).
Biotinylated goat anti-rabbit IgG secondary antibody (Vector Laboratories, #PK-6101) is validated for immunohistochemical analysis, immunocytochemical analysis, in situ hybridization, ELISA; this antibody works rabbit (https://vectorlabs.com/products/abc-kits/vectastain-elite-abc-hrp-kit-rabbit-igg).

Eukaryotic cell lines

Policy information about cell lines and Sex and Gender in Research

Cell line source(s): Human cell line HEK293T was purchased from ATCC (#CRL-3216) .

Authentication: None of the cell lines were authenticated.

Mycoplasma contamination: Cell lines were not tested for mycoplasma contamination

Commonly misidentified lines (See ICLAC register): None to declare

Animals and other research organisms

Policy information about studies involving animals; ARRIVE guidelines recommended for reporting animal research, and Sex and Gender in Research

Laboratory animals:

1. C57BL/6J (WT) mice: ages 7-8 weeks old, age and sex matched, only male mice used.
2. Ssu72 WT mice on C57BL/6J background (Ssu72 flox/flox): ages 6-22 weeks old, age and sex matched, both males and females used.
3. Ssu72 aKO mice on C57BL/6J background (Ssu72 flox/flox; Adiponectin-Cre): ages 6-22 weeks old, age and sex matched, both males and females used.
4. Ssu72 aKO;cTg mice on C57BL/6J background (Rosa26 HA-Ssu72; Ssu72 flox/flox; Adiponectin-Cre): ages 6-14 weeks old, age and sex matched, both males and females used.

All mice are housed under temperature-controlled (23 degrees Celsius) and humidity-controlled (40-60%) conditions with free access to food and water, on a 12-hour light/12-hour dark cycle.
Both male and female mice were used for experiments, and no sex-specific phenotype was observed in vivo.

Wild animals: This study did not involve wild animals.

Reporting on sex: Findings reported in this study apply to both male and female animals.

Field-collected samples: This study did not involved field-collected samples.

Ethics oversight: All animal experiments were conducted in accordance with guidelines of the Institutional Animal Care and Use Committee (IACUC) of Sungkyunkwan University School of Medicine (SUSM), which is accredited by the Association for Assessment and Accreditation of Laboratory Animal Care International (AAALAC International) and abides by the Institute of Laboratory Animal Resources (ILAR) guidelines.
Metabolic studies were conducted at Soonchunhyang University in accordance with guidelines of an approved IACUC of Soonchunhyang Institute of Medi-bio Science (SIMS) protocol (SCH-IACUC).

Note that full information on the approval of the study protocol must also be provided in the manuscript.
